# Efficacy of combination products containing sarolaner, moxidectin and pyrantel (Simparica Trio™) or afoxolaner and milbemycin (NexGard Spectra^®^) against induced infestations of *Ixodes holocyclus* in dogs

**DOI:** 10.1186/s13071-020-04323-8

**Published:** 2020-09-05

**Authors:** Raj Packianathan, Andrew Hodge, Natalie Bruellke, Chrissie Jackson, Steven Maeder

**Affiliations:** 1 Zoetis Australia Research and Manufacturing Pty Ltd, Veterinary Medicine Research and Development, Level 6, 5 Rider Boulevard, Rhodes, NSW 2138 Australia; 2 Invetus, 495 Ellangowan Road, Yorklea, NSW 2470 Australia

**Keywords:** Isoxazoline, *Ixodes holocyclus*, NexGard Spectra^®^, Simparica Trio™

## Abstract

**Background:**

The Australian paralysis tick, *Ixodes holocyclus,* causes tick paralysis in dogs and cats in the eastern coastal regions of Australia. Prevention is the best option to protect dogs against this potentially fatal disease and sarolaner provides rapid and sustained efficacy against *I. holocyclus*. In this laboratory study, the efficacy of two combination endectocides containing sarolaner + moxidectin + pyrantel (Simparica Trio™) and afoxolaner + milbemycin (NexGard Spectra^®^) was evaluated against an artificial infestation of *I. holocyclus*.

**Methods:**

Twenty-four (*n* =24) foxhounds were randomly allocated to three treatment groups and artificially infested with 30 adult female viable ticks on Days − 1, 7, 14, 21, 28 and 35. On Day 0, dogs in each treatment group were treated with either Drontal^®^ (control group), Simparica Trio™ at the label dose to provide minimum doses of sarolaner (1.2 mg/kg), moxidectin (24 µg/kg) and pyrantel (5 mg/kg) or NexGard Spectra^®^ to provide minimum doses of afoxolaner (2.5 mg/kg) and milbemycin (0.5 mg/kg). Live tick counts were performed at 48 and 72 hours after treatment and after each re-infestation on Days 7, 14, 21, 28 and 35. Efficacy was determined at each time point relative to counts for control dogs based on geometric means.

**Results:**

Against an existing infestation, efficacy of both Simparica Trio™ and NexGard Spectra^®^ was 99.6% and 100% at 48 and 72 h time points, respectively (*P *= 1.000). Against subsequent weekly infestations, treatment with Simparica Trio™ and NexGard Spectra^®^ resulted in efficacy of ≥ 97.7% and ≥ 95.5% (*P* ≥ 0.0911), respectively at the 48 h time point and at the 72 h time point, Simparica Trio™ and NexGard Spectra^®^ resulted in efficacy of ≥ 99.0% and ≥ 98.4% (*P* ≥ 0.0511), respectively. There were no treatment-related adverse events in the study.

**Conclusions:**

Single doses of Simparica Trio™ and NexGard Spectra^®^ were highly efficacious and provided comparable efficacy against the Australian paralysis tick, *I. holocyclus* for up to 35 days.
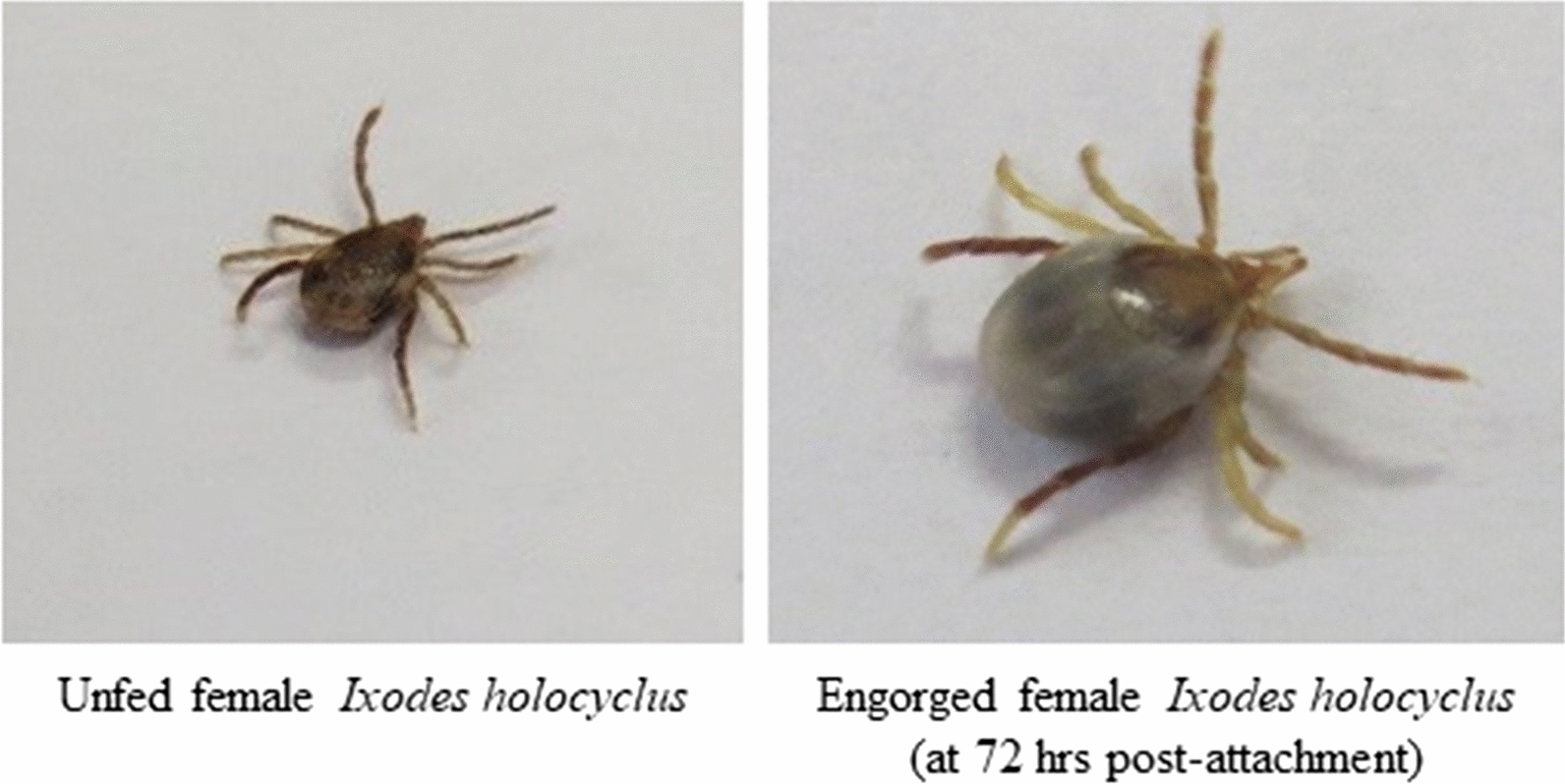

## Background

The isoxazoline class of ectoparasiticides such as sarolaner, afoxolaner, fluralaner and lotilaner are highly efficacious and have broader spectrum activity against ticks, fleas and/or mites [1–9]. The combination of the isoxazolines with one or more additional active ingredients has provided activity against other parasites, such as heartworm and gastrointestinal nematodes [10–14]. Heartworm disease caused by *Dirofilaria immitis* is endemic in Australia [15–18]. Currently, the majority of heartworm cases in dogs are reported in the tropical regions of Australia, such as Far North Queensland [19] where an abundance of heartworm reservoirs and vectors are present [20, 21]. Gastrointestinal nematode infections are one of the most common diseases in dogs and endemic throughout Australia [15, 22–25].

*Ixodes holocyclus*, also known as the Australian paralysis tick, is the major cause of tick paralysis in dogs in Australia. It is widely distributed along the eastern coastal regions of Australia from North Queensland to Lakes Entrance of Victoria [26–29]. Another ixodid tick, *Ixodes cornuatus*, is also reported to cause clinically significant paralysis in some parts of south-eastern Australia [29, 30]. Clinical manifestations of tick paralysis caused by *I. holocyclus* in dogs have been well documented [31, 32]. Treatment of tick paralysis involves removal of attached ticks to prevent further envenomation and administration of tick anti-sera to neutralise the circulating free holocyclotoxins while stabilising the secondary cardio-pulmonary complications [33, 34]. Prognosis of tick paralysis cases depends on various factors such severity of clinical signs at the time of presentation, seasonality and potency of the anti-sera [31, 34]. Prevention is therefore the best option to protect dogs and cats against this fatal disease [35]. Since the introduction of isoxazolines in Australia, the incidence of tick paralysis related insurance claims appears to be declining which is a testimony to their efficacy [36].

These parasites are also zoonotic in nature and can potentially infect humans. Zoonotic diseases caused by *Toxocara canis*, *Ancylostoma caninum* and *Echinococcus granulosus* in humans are well understood [37–40]. Tick paralysis caused by *I. holocyclus* [41] and heartworm infections in humans have also been reported [42, 43]. *Ixodes holocyclus* is also suspected to act as a vector for transmission of *Borrelia burgdorferi* (*sensu stricto*) [44] and *Rickettsia australis* in humans [45, 46]. Hence, these combination products, with a broad spectrum of activity, not only protect dogs against these diseases, but also are important for human health thus supporting the ‘One Health paradigm’ [47, 48].

Here we report on a laboratory study to evaluate and compare the efficacy of two combination endectocides containing sarolaner + moxidectin + pyrantel (Simparica Trio™, Zoetis, Australia) and afoxolaner + milbemycin (NexGard Spectra^®^, Boehringer Ingelheim Animal Health, Australia) against an artificial infestation of *I. holocyclus*. Simparica Trio™ is approved in Australia for the treatment and control of fleas, ticks (*I. holocyclus* and *R. sanguineus*) and gastrointestinal worms (hookworms and roundworms) as well as the prevention of heartworm disease caused by *D. immitis* in dogs.

Sarolaner (Simparica™, Zoetis) chewable tablet dosed at 2 mg/kg body weight, a potent ecto-parasiticide, has excellent efficacy against ticks, fleas and mites [6] and has been demonstrated to provide rapid and sustained efficacy against *I. holocyclus* [49]. To broaden the spectrum of activity, a new novel oral chewable combination product delivering 1.2 mg/kg sarolaner, 24 µg/kg moxidectin and 5 mg/kg pyrantel (Simparica Trio™, Zoetis) has been approved in USA, Canada, Europe and Australia. Simparica Trio™ has been shown to be efficacious against various tick species and fleas in the USA and Europe [50–54].

## Methods

This was a blinded, negative-controlled, randomised laboratory efficacy study conducted in New South Wales (NSW), Australia. Study procedures were in accordance with the World Association for the Advancement of Veterinary Parasitology (WAAVP) guidelines for evaluating the efficacy of parasiticides for the treatment, prevention and control of flea and tick infestation on dogs and cats [55] and complied with the principles of Good Clinical Practice [56]. The study was conducted according to the Australian Pesticides and Veterinary Medicines Authority (APVMA) guidelines [57]. The protocol was reviewed and approved by the Wongaburra Research Centre Animal Ethics Committee, NSW, Australia. Blinding of the study was assured through the separation of functions. All personnel conducting observations, or performing infestations and counts were blinded to treatment allocation.

### Animals

Twenty-four (*n *= 24) pure and crossbred Foxhound dogs of both sexes (14 females and 10 males), aged between 3 and 8 years were enrolled in the study. Each dog was individually identified by a unique electronic transponder. All dogs had undergone an adequate wash-out period for at least 90 days to ensure that no residual ectoparasiticide efficacy remained from any previously administered treatments which was confirmed by the tick carrying capacity tests conducted on day − 8. Dogs were individually housed in indoor runs such that no physical contact was possible between them and they were acclimatised to these conditions for at least 14 days prior to treatment. Dogs were fed an appropriate maintenance ration of a commercial dry canine feed for the duration of the study. Water was available *ad libitum*. All dogs were given a physical examination to ensure that they were in good health at enrolment and suitable for inclusion in the study. General health observations were performed three times daily throughout the study.

### Design

The study followed a randomised complete block design, with pairs of dogs as the experimental unit. Dogs enrolled in the study were immunised to the tick toxin-holocyclotoxin as described previously [58]. Prior to treatment on Day 0, dogs were ranked according to pre-treatment tick counts into four blocks of six (three pairs of dogs). Within each block, one pair of dogs was randomly allocated to one of three treatment groups. Dogs in the control group were treated with praziquantel (175 mg) + pyrantel (174.4 mg) + febantel (875 mg) combination (Drontal^®^ Bayer, Australia) for the control of pre-existing gastrointestinal worm burden, as per the label dose (1 tablet per 35 kg). As Drontal^®^ does not have any acaricidal effect, this group functioned as the negative control group for the assessment of tick counts in this study. Dogs in the other two groups were treated with combination products containing either sarolaner + moxidectin + pyrantel (Simparica Trio™, Zoetis, Australia) or afoxolaner + milbemycin (NexGard Spectra^®^, Boehringer Ingelheim Animal Health, Australia).

### Treatment

Body weights recorded on the treatment day were used to calculate the appropriate dose of each treatment and the enrolled dogs weighed between 28.7 and 42.2 kg. All dogs were fasted overnight prior to Day 0 and the first feed was offered approximately 4 h after treatment administration. Dogs received their respective treatments according to the label instructions. The dogs in the Simparica Trio™-treated group received sarolaner at minimum 1.2 mg/kg (actual doses ranged from 1.30 to 1.71 mg/kg), moxidectin at minimum 24 µg/kg (26.0–34.1 µg/kg) and pyrantel at minimum 5 mg/kg (5.42–7.11 mg/kg). The dogs in the NexGard Spectra^®^-treated group received afoxolaner at minimum 2.5 mg/kg (actual doses ranged between 2.61–4.63 mg/kg) and milbemycin at minimum 0.5 mg/kg (0.52–0.93 mg/kg). All doses were administered by hand pilling to ensure accurate and complete dosing. Each dog was observed for at least 2 min after treatment to ensure the dose was swallowed and for 2 h for evidence of vomiting. Vomiting was recorded only in one dog treated with Drontal^®^ in the study. The dogs were monitored for clinical signs approximately 1, 3, 6 and 24 h post-treatment.

### Tick infestation and assessment

Wild-caught, unfed adult female *I. holocyclus* ticks collected from Queensland and the Northern Rivers region of New South Wales, Australia were used in the study. The ticks were stored in dark conditions at around 12 °C and high humidity [58] for approximately 9 months prior to the start of the study. Prior to each infestation, dogs were examined to ensure they were free of ticks. Each dog was infested with 30 adult female unfed, viable ticks on each of Days − 1, 7, 14, 21, 28 and 35 at pre-defined locations (head, shoulders, dorsal midline of the body and tail base) on the dogs as described previously [58]. Following Day − 1 infestation, tick counts were performed at 48 and 72 h after treatment on Day 0 to evaluate the immediate efficacy of treatment and all other tick counts were performed at 48 and 72 h after each weekly infestation to evaluate the persistent efficacy. Tick assessments at 48 h time points were performed without removing the ticks from the dogs. After counting at the 72 h time point, all ticks were removed. Both free and attached ticks were characterized as either live or dead as described previously [58]. Moribund ticks were characterized and included in the live counts as previously described [49]. All the tick counts and assessments were performed by laboratory technicians who were experienced and trained on validated laboratory methods of tick counting and assessments.

### Statistical analysis

The primary outcome measure was live tick counts. Data for post-treatment live (free plus attached) tick counts were summarised with arithmetic (AM) and geometric (GM) means by treatment group and time point. Tick counts were transformed by the log_e_(count + 1) transformation prior to analysis in order to stabilise the variance and normalise the data. Using the PROC MIXED procedure (SAS 9.4, SAS Institute Inc., Cary, NC, USA), transformed counts were analysed using a mixed linear model for repeated measures for the 48 and 72 h time points separately. The fixed effects were treatment, time point and the interaction between time point and treatment. The random effects included block, pair, animal, block by treatment by time point interaction, and error. Testing was two-sided at the significance level α= 0.05, with tests based on contrasts between treatment least squares means from the fitted models.

The assessment of efficacy for live ticks was based on the percent reduction in the AM and GM live tick counts for the treated groups relative to control, as suggested by the most recent guidelines of the WAAVP for systemic acaricides [35], and was calculated using Abbott’s formula:

$${\text{\% Reduction}} = 100 \times \frac{{{\text{Mean count }}\left( {\text{control}} \right){-}{\text{Mean count }}\left( {\text{treated}} \right)}}{{{\text{Mean count }}\left( {\text{control}} \right)}}$$

As the distribution of parasite counts within each group was likely be skewed, comparison between groups was primarily based on GM live tick counts [55].

## Results and discussion

### Safety

Three dogs in the Simparica Trio™-treated group were treated for dermatitis or lick granuloma and three dogs in the NexGard Spectra^®^-treated group were treated for trauma, lick granuloma and/or gastrointestinal upset. None of these adverse events were considered as treatment-related.

### Efficacy

Dogs in the control group maintained good tick infestations throughout the study with individual tick counts ranging from 16 to 31 and geometric mean counts between 20 to 26 (Tables 1 and 2).

**Table 1:** Mean live *Ixodes holocyclus* counts and efficacy relative to control at 48 h after treatment and after weekly re-infestations for dogs treated with a single oral dose of Simparica Trio™ or NexGard Spectra^®^ on Day 0

Treatment	Day of study					
	2	9	16	23	30	37
Control						
Range	17–27	16–26	22–31	20–28	19–29	18–30
Arithmetic mean (AM)	22.13	20.63	25.38	24.50	23.50	22.75
Geometric mean (GM)^a^	21.92	20.45	25.26	24.40	23.31	22.39
Simparica Trio™						
Range	0–1	0–3	0–2	0–2	0–1	0–2
Arithmetic mean (AM)	0.13	0.63	0.50	0.75	0.13	0.38
AM Efficacy (%)	99.44	96.97	98.03	96.94	99.47	98.35
Geometric mean (GM)^a^	0.09	0.41	0.36	0.57	0.09	0.25
GM Efficacy (%)	99.59	97.97	98.56	97.68	99.61	98.88
Test statistic *vs* control	*t*_(51)_= 19.75	*t*_(51)_= 17.63	*t*_(51)_= 19.18	*t*_(51)_= 18.07	*t*_(51)_= 20.13	*t*_(51)_= 18.99
*P*-value *vs* control	< 0.0001	< 0.0001	< 0.0001	< 0.0001	< 0.0001	< 0.0001
NexGard Spectra^®^						
Range	0–1	0–5	0–4	0–7	0–1	0–4
Arithmetic mean (AM)	0.13	1.13	1.25	1.75	0.75	1.25
AM Efficacy (%)	99.44	94.55	95.07	92.86	96.81	94.51
Geometric mean (GM)^a^	0.09	0.71	0.78	1.10	0.68	0.91
GM Efficacy (%)	99.59	96.54	96.92	95.48	97.08	95.92
Test statistic *vs* control	*t*_(45)_= 14.37	*t*_(45)_= 11.95	*t*_(45)_= 12.71	*t*_(45)_= 11.76	*t*_(45)_= 12.61	*t*_(45)_= 11.82
*P*-value *vs* control	< 0.0001	< 0.0001	< 0.0001	< 0.0001	< 0.0001	< 0.0001
Test statistic *vs* sarolaner	*t*_(73)_= 0.00	*t*_(73)_= 0.74	*t*_(73)_= 1.05	*t*_(73)_= 1.17	*t*_(73)_= 1.71	*t*_(73)_= 1.68
*P*-value *vs* sarolaner	1.0000	0.4599	0.2984	0.2461	0.0911	0.0972

^a^*P*-values are based on comparison of geometric means

**Table 2:** Mean live *Ixodes holocyclus* counts and efficacy relative to control at 72 h after treatment and after weekly re-infestations for dogs treated with a single oral dose of Simparica Trio™ or NexGard Spectra^®^ on Day 0

Treatment	Day of study					
	3	10	17	24	31	38
Control						
Range	17–27	17–27	23–28	21–27	17–29	19–28
Arithmetic mean (AM)	21.88	20.88	25.00	24.25	23.13	23.13
Geometric mean (GM)^a^	21.64	20.69	24.95	24.18	22.86	22.94
Simparica Trio™						
Range	0–0	0–0	0–2	0–0	0–1	0–0
Arithmetic mean (AM)	0.00	0.00	0.38	0.00	0.13	0.00
AM Efficacy (%)	100	100	98.50	100	99.46	100
Geometric mean (GM)^a^	0.00	0.00	0.25	0.00	0.09	0.00
GM Efficacy (%)	100	100	98.99	100	99.60	100
Test statistic *vs* control	*t*_(34)_= 28.97	*t*_(34)_= 28.53	*t*_(48)_= 27.10	*t*_(41)_= 23.66	*t*_(33)_= 17.13	*t*_(36)_= 29.05
*P*-value *vs* control	< 0.0001	< 0.0001	< 0.0001	< 0.0001	< 0.0001	< 0.0001
NexGard Spectra^®^						
Range	0–0	0–0	0–0	0–1	0–2	0–1
Arithmetic mean (AM)	0.00	0.00	0.00	0.25	0.50	0.25
AM Efficacy (%)	100	100	100	98.97	97.84	98.92
Geometric mean (GM)^a^	0.00	0.00	0.00	0.19	0.36	0.19
GM Efficacy (%)	100	100	100	99.22	98.41	99.18
Test statistic *vs* control	*t*_(34)_= 28.97	*t*_(34)_= 28.53	*t*_(48)_= 29.10	*t*_(41)_= 22.39	*t*_(33)_= 15.89	*t*_(36)_= 27.47
*P*-value *vs* control	< 0.0001	< 0.0001	< 0.0001	< 0.0001	< 0.0001	< 0.0001
Test statistic *vs* Simparica Trio™	*t*_(34)_= 0.00	*t*_(34)_= 0.00	*t*_(48)_= -2.00	*t*_(41)_= 1.27	*t*_(33)_= 1.24	*t*_(36)_= 1.59
*P*-value *vs* Simparica Trio™	1.0000	1.0000	0.0511	0.2109	0.2225	0.1217

^a^*P*-values are based on comparison of geometric means

Against an existing infestation, at the 48 and 72 h time points, treatment with Simparica Trio™ and NexGard Spectra^®^ resulted in significantly lower GM tick counts compared to the control group (14.37 ≤ *t*_*df*_ ≤ 28.97 where 34 ≤ *df* ≤ 51, *P *< 0.0001). Against the existing infestation, both Simparica Trio™ and NexGard Spectra^®^ provided 99.6% and 100% efficacy at 48 and 72 h post-treatment, respectively. The comparable efficacy results at the 48 h time point were consistent with those previously reported [49] (Tables 1 and 2). Based on arithmetic mean counts, the efficacy of both Simparica Trio™ and NexGard Spectra^®^ against an existing infestation at the 48 h and 72 h time points was 99.4% and 100%, respectively.

Against subsequent weekly infestations at the 48 h time point, treatment with Simparica Trio™ and NexGard Spectra^®^ resulted in efficacy of ≥ 97.7% and ≥ 95.5%, respectively. At the 72 h time point, Simparica Trio™ and NexGard Spectra^®^ resulted in efficacy of ≥ 99.0% and ≥ 98.4%, respectively. At all time points, both treatments resulted in significantly lower GM tick counts compared to the control group (11.76 ≤ *t*_*df*_ ≤ 29.10 where 33 ≤ *df* ≤ 51, *P *< 0.0001). However there were no significant differences in the GM tick counts between the two treatments at any time point (-2.00 ≤ *t*_*df*_ ≤ 1.71 where 33 ≤ *df* ≤ 73, *P* ≥ 0.0511). Based on arithmetic mean counts, the efficacy of Simparica Trio™ and NexGard Spectra^®^ against subsequent weekly infestations was ≥ 96.9% and ≥ 92.9%, respectively, at the 48 h time points and ≥ 97.8% for both products at the 72 h time points.

Single doses of Simparica Trio™ and NexGard Spectra^®^ resulted in the rapid reduction of an existing infestation and subsequent re-infestations of live *I. holocyclus* ticks for up to 5 weeks. Although the onset of clinical signs of tick paralysis does not occur until 4 or 5 days after tick attachment, the sooner the attached ticks can be killed, the lower the chance of tick paralysis [59–61]. The rapid and sustained speed of kill of Simparica Trio™ after a single oral dose will minimise the risk of tick paralysis. Similar efficacy of Simparica Trio™ has also been demonstrated against ixodid ticks in Europe (*Ixodes ricinus* and *Ixodes hexagonus*) and the USA (*Ixodes scapularis*), respectively [51, 53].

## Conclusions

Single doses of Simparica Trio™, containing sarolaner, moxidectin and pyrantel and NexGard Spectra^®^ containing afoxolaner and milbemycin provided comparable efficacy against the Australian paralysis tick, *I. holocyclus* for up to 35 days. These two combination products with a broader spectrum of activity will provide effective control of the most important parasites in dogs including the Australian paralysis tick, fleas, heartworm, roundworms and hookworms of zoonotic significance, thus offering a holistic one health treatment option to the pet owners.

## Data Availability

The dataset supporting the conclusions of this article is included within the article.

## Abbreviations

AM: arithmetic mean
GM: geometric mean
WAAVP: World Association for the Advancement of Veterinary Parasitology
